# KNOWLEDGE ABOUT THE BENEFITS OF BREASTFEEDING AND DISADVANTAGES OF THE PACIFIER RELATED TO THE MOTHER’S PRACTICE WITH PRETERM INFANTS

**DOI:** 10.1590/1984-0462/;2017;35;4;00005

**Published:** 2017-09-21

**Authors:** Elâine Cristina Vargas Dadalto, Edinete Maria Rosa

**Affiliations:** a Universidade Federal do Espírito Santo (UFES), Maruípe, ES, Brasil.; b UFES, Goiabeiras, ES, Brasil.

**Keywords:** Sucking behavior, Breastfeeding, Pacifiers, Premature birth, Comportamento de sucção, Aleitamento materno, Chupetas, Nascimento prematuro

## Abstract

**Objective:**

To evaluate the knowledge and expectations of mothers of preterm newborn infants admitted in a neonatal intensive care unit about breastfeeding and pacifier use, and to analyze their experience in dealing with the sucking urge in the first months of life.

**Methods:**

Mothers were interviewed during hospitalization of the newborn in the neonatal intensive care unit and when the infant was six months old. All mothers with availability to participate in the study were included. Exclusion criteria comprised infants with syndromes and neurological disorders and mothers with cognitive impairment, depression, and drug users. Data were analyzed with the SPSS software, with descriptive statistics and chi-square test.

**Results:**

Sixty-two mothers were interviewed in the beginning and 52 at a six-month follow-up. Mothers’ expectations concerning breastfeeding were positive when they listed the benefits to the mother (90.3%) and infant (100%). However, they had difficulties maintaining exclusive breastfeeding and used the baby bottle (75.0%), which most mothers (69.4%) had already acquired before the infant was born. The fact of having a pacifier in the infant’s layette (43.6%) did not influence its use (*p*=0.820). This also occurred among mothers who said they would not offer the pacifier due to disadvantages to the mother (80.7%) and infant (96.8%). The previous expectation that the pacifier could bring benefits for mother and infant did not affect its use (*p*=0.375 and *p*=0.158).

**Conclusions:**

Mothers demonstrated prior knowledge about breastfeeding benefits and disadvantages of the pacifiers. However, they changed their view when dealing with the infant and introduced bottles and pacifiers.

## RESUMO

**Objetivo:**

Avaliar conhecimentos e expectativas de mães de recém-nascidos pré-termo (RNPT) internados em unidade de terapia intensiva neonatal (UTIN) sobre aleitamento materno (AM) e uso de chupeta; e analisar sua vivência ao lidar com a necessidade de sucção nos primeiros meses.

**Métodos:**

As mães foram entrevistadas durante a internação dos recém-nascidos (RN) na UTIN e quando eles completaram seis meses de idade. Foram incluídas todas as mães com disponibilidade para participar do estudo. Os critérios de exclusão englobaram RNs com síndromes ou distúrbios neurológicos e mães com comprometimento cognitivo, depressão e usuárias de drogas. Os dados tabulados no programa *Statistical Package for the Social Sciences* (SPSS) foram analisados por estatística descritiva e teste qui-quadrado.

**Resultados:**

Foram entrevistadas 62 mães inicialmente e 52 no *follow-up* de seis meses. As expectativas das participantes quanto à amamentação foram positivas, visto que elas relataram benefícios para mãe (90,3%) e bebê (100%), mas tiveram dificuldades para manter o aleitamento exclusivo, intr oduzindo a mamadeira (75,0%), já adquirida pela maioria (69,4%) antes do nascimento. O fato de haver chupeta no enxoval do RN (43,6%) não influenciou seu uso (*p*=0,820), tendo ocorrido também quando as mães não iriam ofertá-la devido às desvantagens para mãe (80,7%) e bebê (96,8%). A expectativa prévia de que a chupeta pudesse trazer benefícios para mãe e bebê não influenciou seu uso (*p*=0,375 e *p*=0,158).

**Conclusões:**

As mães demonstraram conhecimentos prévios sobre benefícios do aleitamento materno e desvantagens da chupeta; entretanto, elas modificaram sua concepção ao lidar com o bebê, recorrendo à introdução de mamadeira e chupeta.

## INTRODUCTION

The preterm newborn (PTNB) that needs hospitalization in a neonatal intensive care unit (NICU), feeding through gastric tube, may present, therefore, delay in the maturity of the suction function and its activity coordinated with breathing and swallowing, depending on gestational age and weight at birth. For hospital discharge, it is necessary that the newborn (NB), besides obtaining systemic conditions, also recovers the sucking activity so that oral feeding is safe.^1^

The stimulation of non-nutritive suction (NNS) has been publicized to anticipate the beginning of feeding by sucking, aiming at reducing the time of hospital stay.^2^ It is recommended that the stimulation of NNS be conducted with the gloved finger, avoiding artificial nipples, in order to not interfere in breastfeeding (BF).^3^

The importance of BF has been related to the prevention of diseases by mothers and pregnant women, and also to the fact that it is important for the whole development of the infant.^4^ The prior experience with breastfeeding increases the prevalence of success in exclusive breastfeeding (EBF).^5^ On the other hand, despite the knowledge coming from professionals or family members in relation to EBF, it has not been sufficient for the orientations to be followed,^6^ which would be essential considering that the establishment of BF is associated with the lower need of complementary NNS.^7^

The offer of a pacifier to stimulate NNS and its use by children represents a cultural phenomenon developed based on a process of internalizing beliefs and practices created by the previous generations.^8^ However, offering a pacifier is controversial among health professionals, who may recommend or not advise its use also based on personal experiences with a cultural influence.^9^ ^,^ ^10^

Even facing the orientation not to use a pacifier in the neonatal period due to the possibility of interfering in the BF process, which might lead to early weaning,^11^ there is divergence between professionals and researchers. An example can be the recommendation of the use of a pacifier due to its relationship with the lower risk of sudden infant death,^12^ its use in PTNB to stimulate NNS, associated or not with musical sounds,^2^ ^,^ ^13^ or to relieve the pain during invasive procedures conducted with hospitalized NBs.^14^

Mothers and pregnant women believe that the pacifier can cause damage to the development of children, but they do not attribute a relationship between the pacifier and BF.^4^ The pacifier is acquired by families even before the baby is born,^15^ as part of the layette.^16^ The main advantage of the pacifier, from the mothers’ point of view, is to nourish the child.^11^ However, the previous conception of the mother regarding the pacifier can be modified based on her interaction with the NB, as reported in a study^17^ in which about one third of the mothers changed opinion due to the rejection of the infant or because of the need to calm the infant down.

Based on these considerations, the purpose of this study was to assess the knowledge and expectations of mothers of PTNBs hospitalized in NICU about BF and the use of a pacifier; and to analyze the experience of these mothers when dealing with the sucking needs of the infant in the first months of life.

## METHOD

The target population was constituted of mothers of PTNBs hospitalized in NICUs, one public and one private institution, in the city of Vitória (ES), from February to June, 2011. The project began after the approval, report n. 249/10, by the Human Research Ethics Committee from the Center of Health Sciences in Universidade Federal do Espírito Santo, according to Resolution n. 196/96, from the National Health Council.

The parameter for prematurity considered birth at gestational age inferior to 37 weeks,^18^ and the inclusion criteria involved all the mothers of PTNBs who were available to participate in the study. Exclusion criteria were NBs with neurological disorders or syndromes and mothers with cognitive impairment, diagnosed with depression, drug users and those whose NB child would be under the tutelage of the Infancy and Youth Court.

At the initial stage, mothers participated in the study during the period when the NB was hospitalized in a NICU, after being transferred from the high complex sector to the intermediate care unit, going to the NB care in a moderate risk situation. All NBs in this study went through the process of NNS stimulation, using the gloved finger technique, and mothers were assisted by the staff to start BF. The contact of mothers with the hospitalized child was daily, and it could last from morning to evening.

When the infants completed six months of age, all mothers were invited by telephone to participate in the second stage of the study, conducted in a pediatric dental clinic related with a public university. This stage of the study was developed during the follow-up dental appointments of the children.

The design proposed for this study was descriptive, observational, with a qualitative and prospective approach, following up a convenience sample of children aged six months. The instruments used for data collection were specifically elaborated for this study, including semi-structured scripts for the interview. The NICU interview was recorded and contemplated the data collection regarding the demographic and social aspects of the participants, as well as questions about the knowledge of mothers about BF and the use of a pacifier. A set of guiding questions stimulated the mothers to verbalize spontaneously about the benefits or advantages, as well as the damage or disadvantages of breastfeeding and the use of a pacifier for the mother and for the infant. Other aspects were also assessed, such as: if there was previous experience in breastfeeding, what was the mothers’ perception and if the pacifier and the baby bottle were present in the layette. Schooling was classified by the Brazilian education system, corresponding to the elementary, high school and higher education.

In the interview for the second stage, the mother’s experience to deal with the child in the first months of life in relation to BF and the use of a pacifier was assessed. The instrument used had objective questions for the collection of information about the advisement received at hospital discharge as to the infant’s diet, time of EBF time of total BF, including complement, introduction of the baby bottle, types of NNS and offer of a pacifier; and open questions, recorded with a voice recorder. In these questions, mothers were asked to express themselves freely about NNS during breastfeeding, and their guide consisted of the following questions: “Do you let or used to let your child to stay on the breast, even if he or she was not sucking strongly? Why?” and “Was there any change in the way the baby sucked the breast after using the bottle? And after introducing the pacifier?”.

The interviews were transcribed and numbered sequentially. Then, the open questions were categorized by semantic approximation, based on the analysis of the content.^19^ The answers to the items in the interview script were tabularized using the software Statistical Package for the Social Sciences (SPSS), version 18.0 for Windows (SPSS Inc., Chicago, IL, USA), and the data were analyzed by descriptive statistics. Considering that among the NNS habits the introduction of the pacifier is conducted by an adult, the categorical variables about the inclusion of the pacifier in the layette and the evaluation of the mothers about the benefits of the pacifier for the mother and for the infant were compared with the use of a pacifier by infants aged six months, using bivariate analyses to verify the relationship between the variables, using the chi-square test.

## RESULTS

The study included mothers of PTNBs who were present during the hospitalization of their children in the period addressed to the study, except for two mothers who gave up their participation. So, 62 mothers of PTNBs were interviewed. To meet the exclusion criteria, the study did not include 79 mothers of NBs with gestational age equal to or higher than 37 weeks; 36 cases of families who did not live in the metropolitan region, or when the NB was transferred to another hospital, or when it was not possible to get in touch with the mother due to the short period of hospitalization; 12 deaths; 9 PTNBs who were still hospitalized in the high complexity sector when data collection was concluded; 3 PTNBs who were referred for a shelter/adoption, 1 case of a PTNBs with severe neurological impairment; and 4 mothers who did not accept to participate in the longitudinal study.

The age of the participants ranged between 17 and 42 years, with mean age of 28.3±6.9 years. In 41.9% of the cases, maternal schooling corresponded to incomplete elementary or high school, and, in 58.1% of the cases, complete high school or higher education. Of the total number of interviewees, 62.9% were inserted in the work market; the marital status of 87.1% was the stable union; 54.8% were primipara; and 88.7% lived in the capital or in the metropolitan region.

The gestational age ranged from 27.4 to 36 weeks, with an average of 33.5±2.21, being 15 (28.8%) neonates classified as extreme preterms or very premature (<34 weeks), and 37 (71.2%) as late preterms (34 to <37); 51.6% of the NBs were female; 17.7% presented weight at birth <1,500 g, 61.3%, ≥1.500 g, and <2,500 g, and 21%, 2,55 g or more. Time of hospitalization varied from 5 to 180 days, and most (72.6%) remained in the hospital from 5 to 30 days; 45.2% of the NBs were fed by orogastric tube (OGT) for 8 days or more; 45.2% for up to 7 days; and in 9.6% of the cases there was no use of the OGT (feeding was nasogastric tube or BF, alternating with sucking on the glass).

The previous feeding experience was reported by 24 (38.7%) participants, and 79.2% had a positive perception, referring a good or great experience and considering the way the body produces food for the other interesting. It is important to mention that one of the mothers qualified the experience as calm, and also donated milk; however, 20.8% of the interviewees had a negative perception (very difficult of causing pain). For the 38 mothers who had no experience with breastfeeding (61.3%), the NB was their first child (n=34) or the first one was adopted (n=4).

In this interview conducted at the hospital, 69.4% of the participants reported that a baby bottle was already available for the NB (29.0 bought it and 40.3% got it as a present); 8.1% still had not acquired one for lack of time to conclude the layette due to premature labor; and 22.6% had not bought or got one. The pacifier was available for 43.6% of the NBs (17.8% bought it and 25.8% got one); 3.3% had not completed the layette; 22.6% had not bought or got one; and 30.6% reported spontaneously that they did not make this object available because they did not want their children using a pacifier.

The answers to the questions about the benefits or advantages and the damage or disadvantages of breastfeeding and using a pacifier, for the mother and the infant, after the analysis of content, were systematized in items. The options of answers for each question were analyzed by descriptive analysis and are demonstrated in Tables 1 and 2.

**Table 1:**
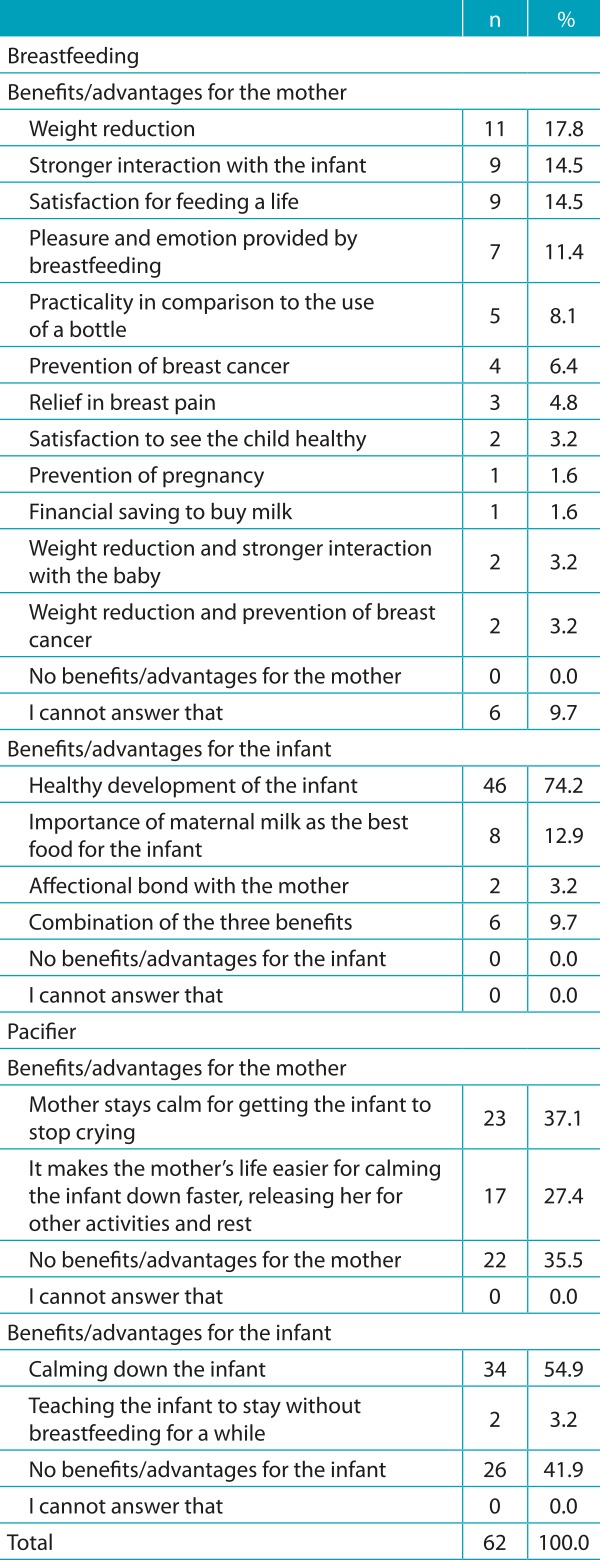
Distribution of participants according to the benefits/advantages of breastfeeding and the use of pacifiers (n=62).

**Table 2:**
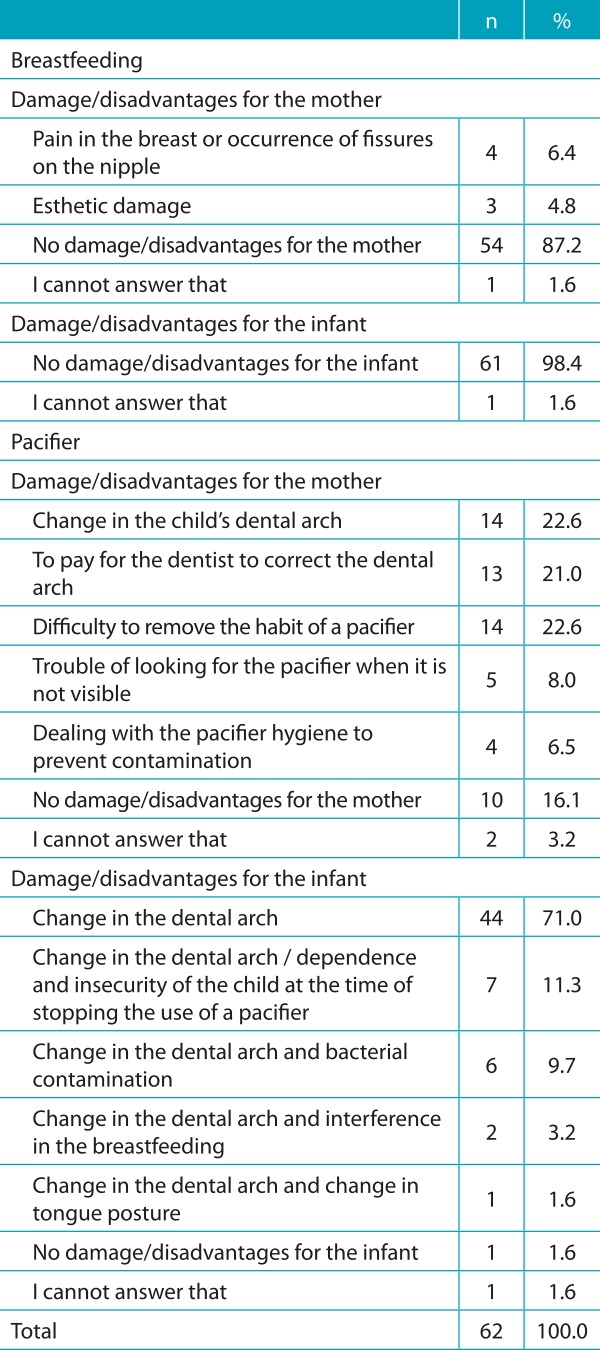
Distribution of participants according to the damage/disadvantages of breastfeeding and the use of pacifiers (n=62).

For the second stage, 2 cases were excluded because the NBs had neurological after-effects during hospitalization, 1 case of death in NICU and 7 mothers who gave up participating in the study due to the difficulty to go to the clinic. So, there were 52 participants, corresponding to 83.9% of the initial group.

In hospital discharge, 76.9% of the mothers were advised for EBF, 11.5% for BF mixed with a supplement in the glass or in the cup, and 5.8% for BF and baby bottle, accounting for 94.2% of BF in the NICU, whereas 5.8% were advised to use the bottle. EBF was reached in 25% of the cases for more than 5 months; 28.8%, from 3 to 5 months; 13.5%, for less than 3 months; whereas for 17.3% there was not EBF, and for 15.4% there was no BF. Considering total BF, including a supplement, 65.4% maintained BF at the age of 6 months (n=34), for 19.2% BF had taken place for at least 62.9% 3 months (n=10), and the other 15.4% did not have BF (n=8). The bottle in the first six months was used by 75.0% of the mothers. In 31 cases there was mixed breastfeeding, and for 61.3% of the mothers there were no changes in the way the infant breastfed after introducing the baby bottle. However, for 38.6% there was a reduction in BF, with lower frequency and lower duration.

At the age of six months, the following types of nutritive and non-nutritive sucking were still present: breastfeeding (n=34, 65.4%), use of bottle (n=39, 75.0%), pacifier sucking (n=26, 50.0%), digital sucking (n=10, 19.2%), tongue ­sucking (n=13, 25.0%), lip sucking (n=5, 9.6%), putting fingers/hand in the mouth (n=52, 100.0%), placing objects/cloth in the mouth (n=42, 80.7%), and sucking on the bottle (n=3, 5.7%).

The use of a pacifier by infants in BF occurred in 19 cases, and for 73.7% of the mothers there was no change in the way the infant breastfed. For 15.8% the time of BF was reduced, and 10.5% reported that infants began to suck the breast harder. Of the mothers who breastfed, 75.8% allowed the infant to continue on the breast even when he or she was not sucking strongly. The expression “the baby uses my breast as a pacifier” was mentioned frequently, referring to the relieving aspect of BF. In the group of mothers whose children used a pacifier, the sense of this phrase for the mother was the release of the NNS component, until then fulfilled by BF, because the infant “stopped using the breast as a pacifier” (interview 11), or “he thought the breast was a pacifier; he only wanted to play” (interview 45). In the group that did not use the pacifier, the expression showed the total uselessness of this object, because the need for NNS was replaced by BF: “Sometimes, I think the breast is a pacifier for him (...) but he uses it as a pacifier, only sleeps on my breast” (interview 31).

The categorical variables about the inclusion of a pacifier in the layette, as well as the expectation of the mothers regarding the benefits of the pacifier - for the mother and the infant - were compared with the use of pacifiers by infants aged 6 months, whose results can be seen in Table 3.

**Table 3:**
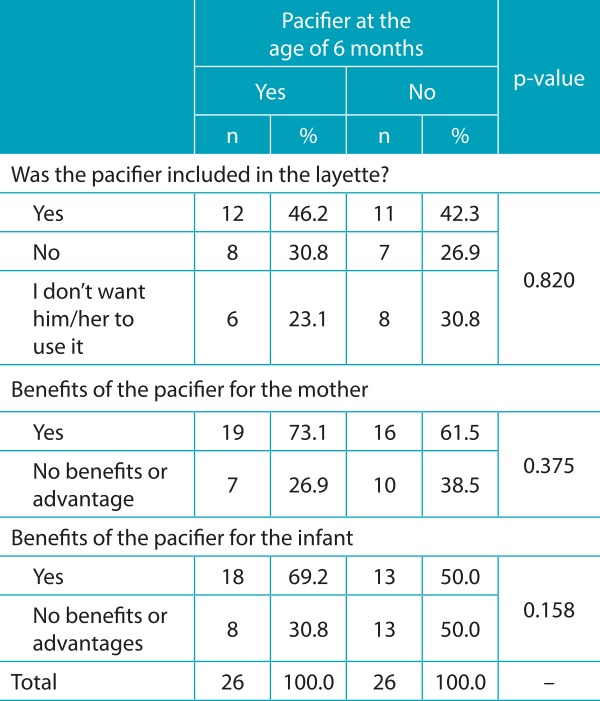
Distribution according to the use of a pacifier by the infant and association with the variables related with the previous expectations of mothers about the pacifier (n=52).

## DISCUSSION

The expectations of participants about BF were very positive, once most of them reported benefits for both the mother and the infant, not seeing any disadvantages. This corroborates with the results presented in the literature as to the high number of benefits for both^20^ and to the perception of BF advantages to prevent diseases and for the development of the infant.^4^

However, it is possible to observe that the psychological benefits for the mother were little explored by the participants of the study, being limited to the pleasure and emotion or to the satisfaction of feeding a child. Besides, there is the need for further studies in this area, addressed, for instance, to the importance of breastfeeding as a protective factor against postpartum depression.^21^

The population was mostly comprised of mothers of late PTNBs, with low weight at birth, who were hospitalized in a NICU up to 30 days and were fed via OGT. Even the late PTNB is metabolically immature, and more vulnerable to intercurrences in the neonatal period in comparison to the term NB,^22^ so, considering this population of biological risk due to the preterm birth and hospitalization in NICU, the results of this study regarding EBF are similar to those of a research in which the frequency of EBF in infants aged up to 2 months was 56.0% in PTNBs, and 75.0% in those born at term.^23^

The use of artificial nipples should be prevented so that it does not interfere in BF,^3^ which is in accordance with the recommendations received by the participants in the period of hospital discharge for the NB, to keep EBF or to use the glass technique in cases of supplementation. However, mixed breastfeeding was very common, and most interviewees acquired a baby bottle before the child was born. Most mothers did not have previous experience in breastfeeding, which would have been a positive aspect to increase the success of BF.^5^

The fact that the pacifier is included in the NB layette (43.6%) was also reported by 49.3% of the participants in another study.^16^ The change in the previous evaluation of the mother as to the offer of a pacifier based on the interaction with the child, already mentioned in the literature,^17^ was also observed in this study, since the introduction of a pacifier occurred both in cases in which mothers had got or bought the pacifier and among those who did not own one, including the ones who had claimed to not want the child to have such an object. The use of a pacifier was also observed when the expectation of the participants was that its use brought no benefits or advantages to the mother and the infant.

The main benefit attributed to the pacifier would be to calm the infant down, and, as a consequence, to facilitate the mother’s routine, in accordance with data from another study.^11^ There was no association between the use or not of a pacifier and variables related with the previous expectations of mothers as to the presence of a pacifier in the layette, and the benefits for the mother and the baby, probably because the change in opinion occurred in both groups (use and non-use of a pacifier), considering the object may have been rejected by the NB or the mother could have interpreted there would be the need to introduce the pacifier as a soothing object.^17^

In most cases, when the infant used a pacifier in BF, the mothers did not report any change in the way the infant sucked the breast, corroborating with findings from another study in which most mothers did not see problems in the relationship between the pacifier and BF.^4^ It is worth to mention that mothers presented positive expectations regarding BF, when interviewed in the NICU, by considering the expressive number of those who listed benefits and advantages of BF, similarly to the positive perceptions of BF expressed by mothers of children aged up to six months.^24^ This result is in accordance with the literature, which leads to the possibility that the interference of the pacifier associated with the early interruption of BF takes place in a specific group of mothers who face difficulties to maintain BF, or are little motivated, not affecting self-confident mothers.^25^

One of the points to analyze and guide mothers regards the need of NNS of the infant during the BF process. Among the mothers who breastfed, most reported allowing the infant to continue the process of BF even when he or she was not sucking strongly, which suggests a pattern compatible with NNS. Nutritive sucking is organized in an uninterrupted and cyclic sequence of movements in medium pace, of one suction per second, whereas NNS comprehends a faster pace, of two suctions per second, and involves the alternation between periods of blast and rest;^26^ it is important to mention that this sucking pace may change in PTNBs.^27^ However, NNS as a need of the infant has not always been understood by mothers in this study, once the expression “uses the breast as a pacifier” was mentioned frequently, meaning that a restrictive action would be necessary, especially among those who introduced the pacifier. Besides, the sucking urge was not cited among the benefits of breastfeeding. To satisfy the need for NNS, the infant may use tongue, lip, hand, finger and object sucking, according to the persistence verified at the age of six months. The suction of objects, cloth, hands and pacifier was an action allowed naturally by the mothers. The exception occurred for digital sucking, and the mother interfered when the practice was constant.

The problems regarding BF may be prevented if professional support is provided for infants after hospital discharge,^28^ with strategies that aim at increasing the duration of EBF,^29^ considering that BF does not only depend on the biological conditions of the NB, but on the maternal reactions facing the challenge of being a mother.^23^ The context should be emphasized, especially because the mothers in this study faced the stress of hospitalizing their children, requiring assistance from a professional encouraging the interaction mother- infant for the success of BF,^1^ ^,^ ^30^ reducing the additional need of NNS. The motivation of mothers should occur after the suggestion of alternatives to deal with the need for sucking, respecting the beliefs and cultural values.

One of the limitations of this study is the small number of participants in a convenience sample, so it is not possible to make a population inference. For further studies, it is suggested to use the sampling calculation with stratification per gestational age, to discriminate the evaluation regarding extreme preterm. One matter that can be explored in future studies refers to the knowledge of mothers about BF facing a situation of prematurity; another important point relates to the relation of the topics studied considering the mothers of infants born at term.

Based on the results presented, it is possible to conclude that mothers of PTNBs demonstrated to have previous knowledge about the benefits of BF and the disadvantages of the pacifier. However, the mothers changed their conception when dealing with the infant by using the introduction of a baby bottle and a pacifier. The fact of having a pacifier in the baby’s layette, as well as the previous expectation that the pacifier could cause benefits for the mother and the infant, did not influence the use at the age of six months. It also occurred when mothers reported they would not offer it due to their preliminary analysis about the disadvantages.
